# Evaluating the Impact of Computerized Provider Order Entry on Medical Students Training at Bedside: A Randomized Controlled Trial

**DOI:** 10.1371/journal.pone.0138094

**Published:** 2015-09-14

**Authors:** Maxime Wack, Etienne Puymirat, Brigitte Ranque, Sophie Georgin-Lavialle, Isabelle Pierre, Aurelia Tanguy, Felix Ackermann, Celine Mallet, Juliette Pavie, Hakima Boultache, Pierre Durieux, Paul Avillach

**Affiliations:** 1 Biomedical informatics and public health department, HEGP, AP-HP, Paris Descartes University, Paris, France; 2 INSERM UMR S 872 team 22, Faculté de Médecine, Paris Descartes University, Paris, France; 3 Departement of Cardiology, HEGP, AP-HP, Paris Descartes University, Paris, France; 4 INSERM U-970, Faculté de Médecine, Paris Descartes University, Paris, France; 5 Department of Internal Medicine, HEGP, AP-HP, Paris Descartes University, Paris, France; 6 Department of Clinical Immunology, HEGP, AP-HP, Paris Descartes University, Paris, France; 7 Department of Infectious Diseases, Saint-Louis Hospital, Paris Diderot University, Paris, France; 8 Centre d’Investigation Épidémiologique 4, INSERM, Faculté de Médecine, Paris Descartes University, Paris, France; 9 Center for Biomedical Informatics, Harvard Medical School & Children’s Hospital Informatics Program, Boston Children’s Hospital, Boston, Massachusetts, United States of America; University of Groningen, University Medical Center Groningen, NETHERLANDS

## Abstract

**Objective:**

To evaluate the impact of computerized provider order entry (CPOE) at the bedside on medical students training.

**Materials and Methods:**

We conducted a randomized cross-controlled educational trial on medical students during two clerkship rotations in three departments, assessing the impact of the use of CPOE on their ability to place adequate monitoring and therapeutic orders using a written test before and after each rotation. Students’ satisfaction with their practice and the order placement system was surveyed. A multivariate mixed model was used to take individual students and chief resident (CR) effects into account. Factorial analysis was applied on the satisfaction questionnaire to identify dimensions, and scores were compared on these dimensions.

**Results:**

Thirty-six students show no better progress (beginning and final test means = 69.87 and 80.98 points out of 176 for the control group, 64.60 and 78.11 for the CPOE group, p = 0.556) during their rotation in either group, even after adjusting for each student and CR, but show a better satisfaction with patient care and greater involvement in the medical team in the CPOE group (p = 0.035*). Both groups have a favorable opinion regarding CPOE as an educational tool, especially because of the order reviewing by the supervisor.

**Conclusion:**

This is the first randomized controlled trial assessing the performance of CPOE in both the progress in prescriptions ability and satisfaction of the students. The absence of effect on the medical skills must be weighted by the small time scale and low sample size. However, students are more satisfied when using CPOE rather than usual training.

## Introduction

According to the Medical School Objectives Project (MSOP), established in January 1996 by the Association of American Medical Colleges to guide medical education, clinical experience is essential in creating memory connections between pathophysiological knowledge and clinical practice, enabling the student to reason flexibly around a case.[1] Bedside practice is critical during medical training as it creates experience in caregiving, diagnosis and therapeutics, and enhances the acquisition of knowledge.[1,2] This is supported by the finding that repeated written testing and testing with Standardized Patients (SPs) in simulations can enhance long-term retention of knowledge, suggesting that the same occurs with testing with real patients.[3]

The widespread of Electronic Health Records (EHRs) and particularly of computerized provider order entry (CPOE) tools in University Hospitals has made them available to students during their clerkship rotations. Students have been showing a greater interest and familiarity with them than attending physicians, independently of computer literacy.[4–6] Although CPOE can facilitate medical prescription and reduce serious medication error rates by more than half [7–12], few studies have addressed the impact of CPOE on the students’ medical training and ability to place sound orders, despite the indication that it could be useful, particularly through the immediate feedback about the placed order.[13–18] These trials yielded negative results, yet they provide insights on the possible causes of failure: lack of randomization, of time and investment from the supervisors, focus on a specialty, factors of confusion such as the addition of learning material in both groups, or a method of evaluation which did not capture the beneficial effect of the intervention.

We hypothesize that *in vivo* use of CPOE can improve medical students training and ability to formalize orders through deeper involvement in the medical practice, that an early contact with it enhances satisfaction and familiarization, and reduces errors. To assess this we conducted an educational randomized cross-controlled trial on medical students in a University Hospital.

## Materials and Methods

The Georges Pompidou European Hospital is a 700 beds university hospital located in Paris, France. It is a fully computerized hospital since its opening in 2000. Students are allowed to place orders on real patients during their clerkship rotations, but these orders are systematically reviewed by a senior MD, usually the Chief Residents (CR) before delivery, being blocked by default until approval. Although this system is available in the whole hospital, it is not yet widely used for teaching purposes, as it requires additional supervising work for the CRs to supervise the students and provide feedback.

We recruited CRs in three departments: Cardiology, Immunology and Internal Medicine, known to provide students good training in placing therapeutic orders. The active and willing participation was a key process in the intervention as it demanded letting the students place their own orders first, dedicating time to review the orders with the students and providing feedback and corrections. Medical students in their last undergraduate year would be included during their clerkship with these CRs in two quarter rotations from January 2012 to June 2012. The randomization was done by CR and students were once and for all assigned to a CR at the beginning of their rotation. Using a cross-over design over these two rotations, CRs were able to be their own controls. The intervention group made use of CPOE at the bedside to place real monitoring and therapeutic orders, and after the patient rounds would discuss the orders with the CR, who would provide corrections and recommendations about the placed orders. As they were actual orders, students needed to specify the drug name, posology, route of administration, hours of delivery and delivery period. The control group didn't use CPOE and bedside teaching was conducted as usual, asking verbally for what the student would do in the current situation. This requires less formalization of the order and vague answers are usually accepted (only the drug class, approximate dosages, etc.)

Eight willing CRs in the three departments were recruited to participate in the study. 36 medical students in their last undergraduate year were included during their clerkship with these CRs over the two quarters of this study. Details of the distribution of CRs and students can be found in Table 1.

**Table 1:** Cross-over schema and distribution of subjects.

Quarter	Intervention	Department	CR	Students
1	Control	Cardio	2	2
1	Control	Int Med	2	4
1	Case	Int Med	2	5
1	Case	Immuno	1	7
2	Case	Cardio	3	3
2	Case	Int Med	2	5
2	Control	Int Med	2	3
2	Control	Immuno	1	7

The primary outcome measure was an increase of knowledge by the students. To evaluate it the students took a 57 items short open answers questionnaire (QROC, S1 Text). Those questions were created independently by three CRs (EP, BR, IP). Two students not included in the study took the test to check the quality of the questions. After validation, all students in the study took this test at the beginning and at the end of the three months period, without any communication of the grades or answers, so as to prevent memory bias. They had one hour to complete them. These questionnaires focused on the ability to prescribe laboratory and imaging diagnosis tests, monitoring and medications in medical situations frequently encountered in the three aforementioned specialties and were rated by two independent MD, PhD (EP and BR) blindly of the department, the student and the group, using a keyword based scale.

The students also filled a 27 items satisfaction questionnaire (S2 Text, adapted from Knight 2005 [17]) at the end of the rotation, consisting of five parts: I) the general opinion of students in regard to placing orders, either on paper or using the computer, II) how they felt about their contribution to the patient’s care, III) the satisfaction with the level of commitment they were allowed to during their clerkship rotation, IV) the obstacles they encountered in placing orders and V) their preferences relating to orders. Part I and III used five points Likert scales ranging from “strongly disagree” to “strongly agree”. Part II used a five point scale from “poor” to “excellent”. Part IV used a four point scale from “no” to “a lot”. In the last part (V) students were asked about the percentage of initial orders they placed for incoming patients, the total percentage of orders they would have liked to place for all their patients, and the percentage of orders they would like to review with their resident, CR and a potential other supervisor.

The orders placed during the first quarter by the students and the associated correction, if any, were extracted from the CPOE database for analysis of the cause of correction.

We checked for any discrepancy in the ratings (grades above the maximum for example) and found none. We then conducted an assessment of inter-rater agreement for the two raters using intra-class correlations coefficients (ICC), both on individual questionnaires and on the students’ progress between the first and the second ones. A first bivariate analysis compared the progress between the case and control group using a two-sided t-test.

A second more detailed analysis used a mixed model to compare the progress of students between the two groups. The model studied the relationship between the progress and: the intervention state as a fixed effect, the item number, the chief resident and the student as random effects. Model selection was done using ANOVA on log likelihood between the different models, choosing the most parsimonious model. p values for the fixed part were calculated using a type 3 hypotheses F test and likelihood ratio tests for the random part.

Items scores in the satisfaction questionnaire were normalized between 0 and 1. We proceeded to do an exploratory factor analysis using the minres factorial method. The number of dimensions was determined using parallel analysis and items were attributed to a dimension based on their loading. Scores for each dimension were obtained by adding the normalized scores from the corresponding items. Bivariate analysis was used to compare scores on all the found dimensions between the two groups.

Statistics were calculated using R version 3.1.1, the lme4 and lmerTest packages for the fitting of linear mixed models[19], and the psych package for psychometric analysis.

### Ethics Statement

This study was reviewed and approved by IRB #00001072 Comité de Protection des Personnes Ile de France II. All Students signed a written informed consent. The study was approved by the Medical faculty Dean of Paris Descartes University.

## Results

36 students in their last undergraduate year were recruited during the two rotations, with a mean age of 24.1 (+/- 0.8) and a 2:1 (female:male) sex ratio. One subject in the second quarter, Cardiology group, has been censored for two reasons. The first one was due to a large inconsistency in grades between examiners. The second one being that this subject was assigned to a CR which didn’t participate in the first quarter. This left us with 35 students total, 18 in the first quarter, 17 in the second one, with the same seven Chief Residents for each quarter.

ICC(3, k) between the two examiners for each item of each questionnaire was 0.93 before censoring, and 0.94 after. ICC(3, k) between the two examiners for the difference between t1 and t2 for each item of the questionnaire was 0.87.

**Fig 1:**
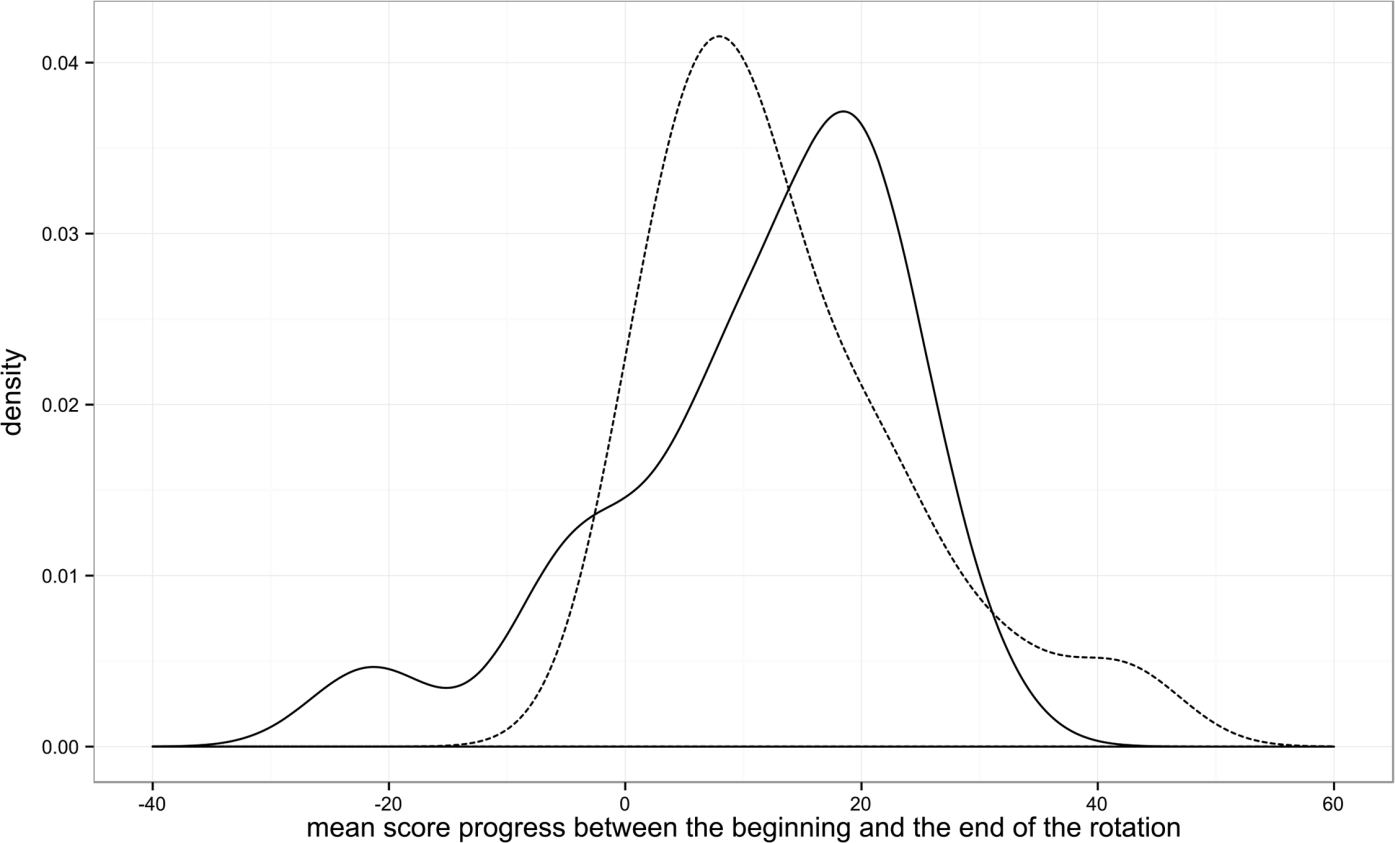
Density plot of the overall questionnaire progress between the beginning and the end of the rotation, for each group. Controls are represented with a continuous line, Cases with a dotted line.

**Fig 2:**
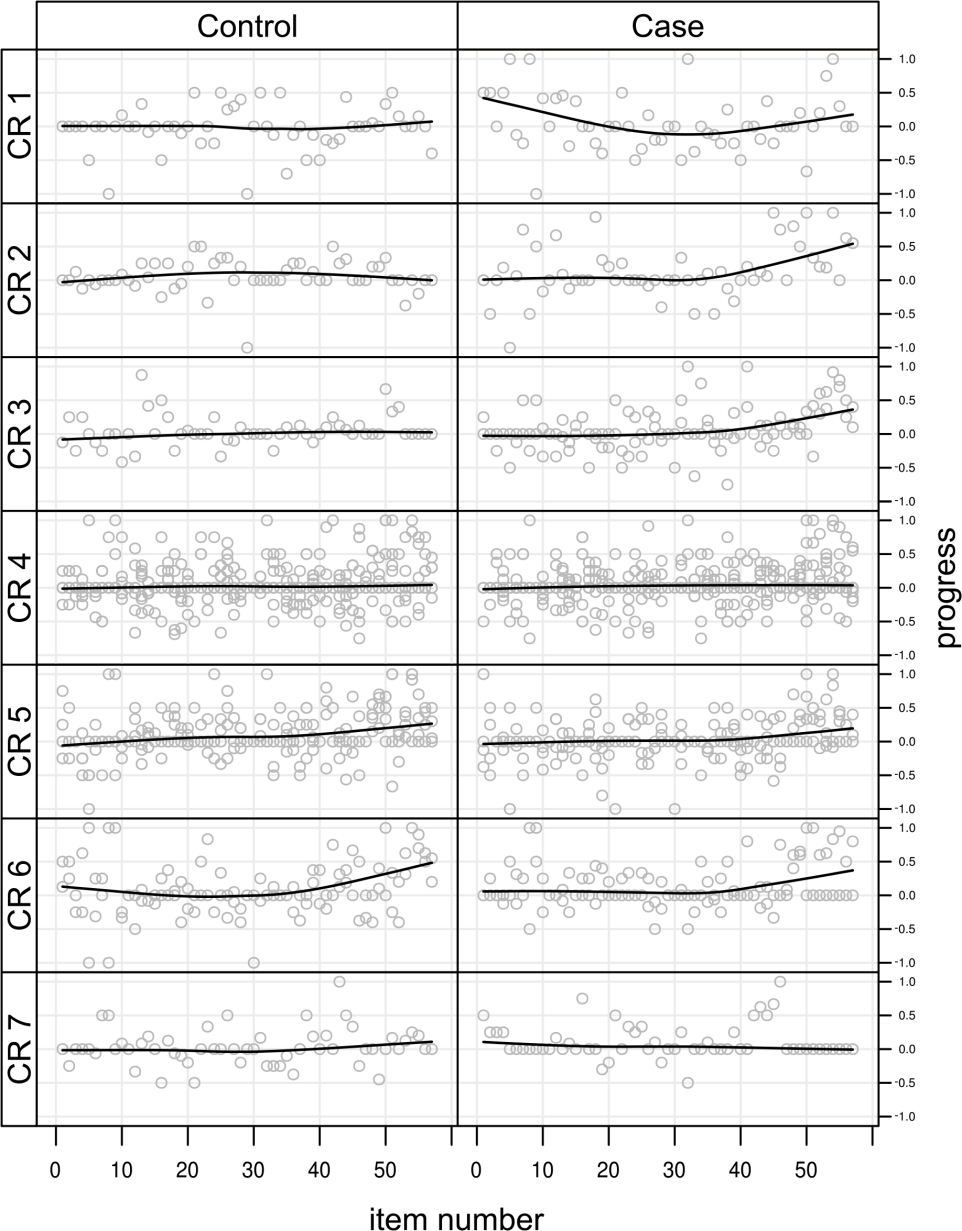
Scatterplots of the progress of the students on each item of the questionnaire between the beginning and the end of the rotation. Plots are shown with one row for each chief resident, left column for the Control group, and right column for the Case group. Item scores have been normalized. A smoothed estimator is fitted for each subplot.

**Fig 3:**
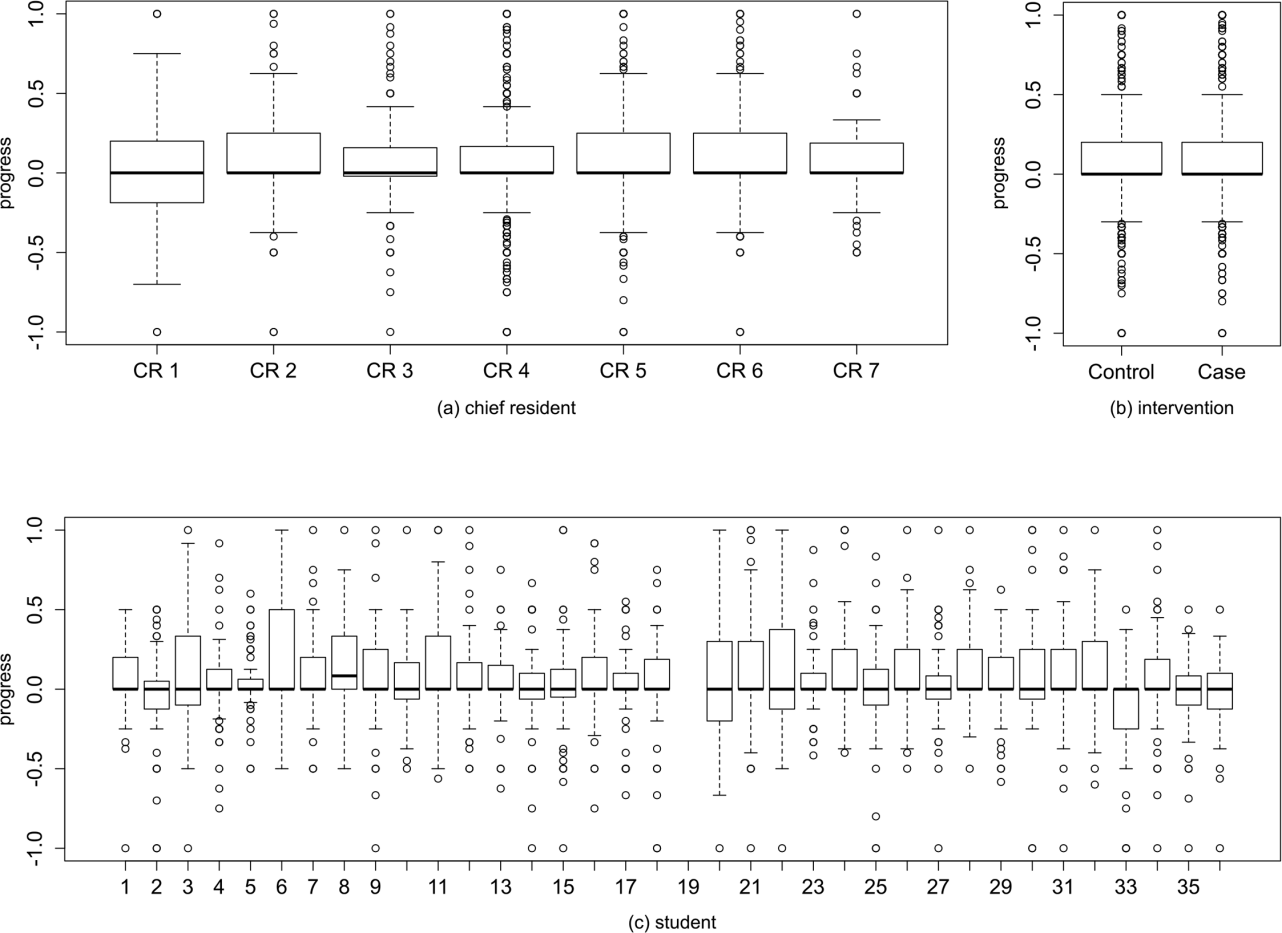
Boxplots of the progress distribution on all questionnaire items for each (a) chief resident, (b) intervention group, (c) student.

The control group scored 69.87 points on the test at the beginning of the rotation, and 80.98 points on the test at the end of the rotation. The case group scored 64.60 points and 78.11 points on the same tests. The highest possible score for these tests was 176. The bivariate analysis shows no statistically significant difference in the progress between the two groups (+11.11 points for the control group, +13.51 points in the case group, p = 0.5562). Overall the students showed a mean progress of +12.35(±11.79) points, significantly different from 0 (4.78e-7). The density plot by group for the overall grade progress between the beginning and the end of the rotation (averaged between the two raters) is shown in Fig 1. Most of the students show a similar progress between the two measures, but one can observe a negative tail for the Control group and a positive tail for the Case group. Fig 2 shows a more detailed representation of the progress (delta in score) of students on individual questionnaire items between the beginning and the end of the rotation. Fig 3 shows the boxplots of the progress (delta in score) for each chief resident (a), intervention group (b) and individual student (c).

The mixed effect model finds a positive yet not statistically significant effect of the Case (vs Control) intervention state, with an estimate of 0.005 (p = 0.82). The random effect associated to the CR has an intercept of 0.0003 (p = 0.60). The random effect associated to individual items of the questionnaire has an intercept of 0.0079 (p = 3.27e-19) and the effect for individual students an intercept of 0.0027 (p = 3e-05).

Other models including a random slope for the effect associated to CRs or a fixed effect for the department didn’t fit the data significantly better, thus we kept the most parsimonious model.

The exploratory factor analysis of the satisfaction questionnaires identified 4 dimensions. The first one corresponds to the overall satisfaction with order practice during the rotation, including 14 items: every item from parts II and III, and the first three items of part IV. The second dimension, including 7 items, reflected the students’ opinion about the usefulness of prescribing in their training. The third dimension (8 items) showed students’ agreement with the benefits of CPOE, and the fourth one (6 items) the difficulties they encountered with it. Representative items in decreasing order of loading for each dimension are listed in Table 2.

**Table 2:** Representative items in decreasing order of loading for each of the four dimensions.

**Overall satisfaction with order practice during the rotation**	III_7: I am receiving adequate preparation for being an intern
**Overall satisfaction with order practice during the rotation**	III_4: My chief resident thought it was important for me to have x
**Overall satisfaction with order practice during the rotation**	III_2: I was included in discussions about the management of my patients
**Overall satisfaction with order practice during the rotation**	IV_1 (negative): Resident or intern did not want me to write or enter orders
**Overall satisfaction with order practice during the rotation**	II_1: During this rotation, what was, in your opinion, your contribution to the medical care of patients?
**Opinion about the usefulness of prescribing in their training**	I_2: Placing orders is an important way to learn what tests and treatments are needed by patients with certain problems
**Opinion about the usefulness of prescribing in their training**	I_1: Placing orders is an important way to increase my sense that I am a caregiver for my patients
**Opinion about the usefulness of prescribing in their training**	V_3: What percentage of your patients total number of admission and follow-up orders would you like to review with your supervisor
**Opinion about the usefulness of prescribing in their training**	I_9: Medical students should be given as many opportunities as possible to place orders
**Opinion about the usefulness of prescribing in their training**	I_10 (negative): The ordering method used will have an impact on my selection of the location of my future rotations
**Agreement with benefit of CPOE**	V_1: For what percent of newly admitted patients that you picked up have you entered the complete set of admission orders?
**Agreement with benefit of CPOE**	IV_10 (negative): Computer ordering system difficult to use
**Agreement with benefit of CPOE**	I_5: Writing orders by hand encourages medical errors
**Agreement with benefit of CPOE**	I_7 (negative): Entering computerized orders encourages medical errors
**Difficulties encountered with CPOE**	IV_4–6: It took too long for the resident/chief resident/other senior to review the orders I wrote
**Difficulties encountered with CPOE**	IV_9: Inadequate training on the computer ordering system
**Difficulties encountered with CPOE**	IV_7: Difficulty in finding a free computer terminal

Scores in the second and third dimensions were normally distributed and compared using Student’s t test. Scores in the first and fourth dimension were not normally distributed and compared using the non-parametric Mann-Whitney U test. They were not different for the second dimension, and were significantly higher for all the other dimensions. Results are shown in Table 3.

**Table 3:** Comparison of dimension scores between the case and control groups.

	Control group		Case group		p value
Dimension	Mean	SD	Mean	SD	
Overall satisfaction (14 items)	6.6	2.8	8.4	3.1	0.035*
Opinion about prescribing (7 items)	4.2	1.5	5.1	1.2	0.057
Opinion about CPOE (8 items)	3.1	0.8	4.3	1	<0.001*
Difficulties with CPOE (6 items)	1.3	1.3	2.4	1.6	0.019*

During the first rotation of this trial, 11 students in the Case group placed 1193 orders, ranging from 28 to 201 (mean = 108.5 +/- 53) per student. 36 (3%) of these orders were urgent orders for laboratory tests or imaging examination and were not reviewed at first by the supervisor, and 97 (8%) were cancelled by the students themselves, leaving 1060 orders for review. 781 orders were for laboratory tests (71 +/- 33.5 per student), of which 108 were corrected, 188 for drugs (17.1 +/- 9), with 43 corrected and 102 for imaging examinations (9.3 +/- 8.2), with 18 corrected. Other kinds of orders included nursing (56), clinical monitoring (34), IV drugs (25), and consultations (7). A total of 202 (19%) orders were corrected during the review process, including 49 orders for oral and IV drugs. The average lag between order placement by students and validation/correction by the supervisor was 1h25 +/- 1h01. A semi-quantitative analysis of the 49 drug prescription errors corrected by CRs is presented in Table 4.

**Table 4:** Drug order errors corrected by Chief Residents.

Type of modification by the supervisor of erroneous orders	Number
Cancellation	9
Modification of dose	7
Modification of treatment duration	5
Modification of drug in the same therapeutic class	5
Modification of the time of delivery	4
Modification of the route of administration and dose (intravenous)	3
Modification of the time of delivery and dose	3
Modification of dose and treatment duration	2
Modification de the time of delivery and dose (intravenous)	2
Modification of the route of administration	2
Modification of drug in another therapeutic class	2
No modification	2
Cancellation because it was already prescribed	2
Modification of individual doses without changing the daily dose	1
**Total**	**49**

## Discussion

This study is the first educational randomized control trial with students prescribing on real patients. Overall the students showed progress in prescription skills between the beginning of the rotation, however no differences were observed between the two groups. After adjusting for questionnaire item, CR, and student, the progress was still not significant between the two groups. Although we thought the CR involvement would be crucial to the students’ progress, due to the nature of the intervention, the multivariate model shows that individual items and students baseline levels are more important. The absence of significant effect of CR on the students’ progress could be explained by the fact that the CRs were all voluntary and already particularly involved in students’ education. We can also hypothesize that the use of CPOE as an educational tool doesn’t require new pedagogical skills from the CRs and only a change of practice. If we can’t rule out a lack of power due to a relatively small sample, the small progress in prescription skills could be explained by the small time scale (3 months) and the fact that participating students were in their last year of undergrad, when little progress can be made before the residency. This could be a limitation of our study, since it induces a lack of power to observe a difference of progress between the two groups.

All the questions from part II and part III of the questionnaire (feeling about the contribution to the patient’s care and satisfaction with the level of commitment they were allowed to) fell expectedly in the same dimension (overall satisfaction with order practice), as their subject is tightly related. Students in the intervention group scored better in this dimension, showing that the use of the actual tools with real patients increased their sense of belonging to the medical team and taking part in patients’ care. Scores for both groups were high in the second dimension (opinion about prescribing), indicating that students see placing orders as an important feature in medical learning, independently of having used paper or computer, albeit nearly reaching statistical significance. It is notable that students who showed a positive opinion about prescribing also wanted to have more of their prescriptions reviewed by a supervisor (correlation coefficients ranging from 0.15 to 0.54 between the corresponding items), showing that not only do they see prescribing as important but also that feedback is essential for the prescribing to be beneficial. The process of reviewing is facilitated by the CPOE as CRs can review the orders in an asynchronous fashion and from different places.

The third dimension included items regarding to the perceived benefits of CPOE by the students. The intervention group also scored higher in this dimension, although they scored higher in the fourth dimension too (difficulties they encountered with CPOE). As they were the only group to use CPOE it is not surprising that they were more aware of its technical issues. However it is still interesting to see that students being exposed to CPOE showed a better opinion toward it than the other group, showing that it met their expectations. The third dimension also contained items V_1 (“For what percent of newly admitted patients that you picked up have you entered the complete set of admission orders?”) and V_2 (“What percentage of your patients’ total number of follow-up orders would you like to write or enter?”), indicating that students who were able to enter more orders perceived more benefits from the use of CPOE, although no causation link can be inferred. It could be indeed that students seeing CPOE use as beneficial were more likely to be confident enough to place orders. Nonetheless, both these items scored significantly better in the CPOE group: 0.01 (+/-0.03) vs 0.1 (+/-0.2), p = 0.032* (item V_1)), and 0.2 (+/-0.3) vs 0.6 (+/-0.4), p = 0.0051* (item V_2).

Students using the CPOE system in the first part of the study mostly ordered laboratory or imaging tests and oral drugs. Although nearly a quarter of all the drug orders needed to be corrected, analysis of these corrections shows that only small adjustments were made by the CRs, such as modifications of the dose or time of delivery.

A first observational study in 1993 following the introduction of a CPOE system in an academic medical center focused on the time spent on the computer placing orders, regarding this time as lost for education. It highlighted the need for an involvement of the attending physicians in the student training of CPOE use.[14] The lack of time to make use of these tools that was reported in other studies might be only a temporary adjustment to a change in routine. If at first reviewing students order can be time-consuming, the integration of this method in common practice could speed up time as well as prepare students to tools they will be using in their future.

In 1995, a randomized controlled study assessed the quality of a fictitious order for an imaginary patient by comparing the orders between the beginning and the end of the rotation and found higher yet non-significant progression in scores in the group which used a computerized system.[15] We found the same not significant result in our study, where actual orders for real patients were placed. In 2001, a non-randomized controlled trial in surgery wards compared the same outcome in questionnaires about surgical procedures, providing additional educational material beside the use of computerized order entry and found no difference between the two groups.[16]

Two non-randomized controlled studies in 2005 and 2012 compared the self-assessed skills and the orders quality between the two groups and found no statistically significant differences. However, students in the CPOE groups reported less occasions to place orders as their supervisors either placed the orders before them or didn’t let them by lack of time.[17,18] In our study design, as the software was already part of the routine workflow in the hospital, CRs were keener on letting students place orders using CPOE. Moreover we used a randomized cross-controlled design to avoid the bias of getting the most motivated students and CRs in the case group as well as a method of analysis taking account of the various uncontrollable factors. The most serious limitation to the cross-over design is that we couldn’t include the same students in the two quarters. However, as the intervention involved two parts we could still preserve comparable entities by randomizing on chief residents. Students showed a greater satisfaction, feeling of commitment to the patients’ care when they used CPOE compared to verbal orders, and reported more occasions to place admission and follow-up orders. We can’t however exclude the possibility of a Hawthorne effect in this case, the students knowing they were observed and in the CPOE group could have felt more motivated.

In departments willing to let students make orders, they are able to do so in a supervised fashion with their orders being blocked until approval by a supervisor, ensuring the patients safety. Chief resident and residents can validate the orders and comment back on them to the students, and although this practice is time-consuming it allows for asynchronous teaching. As the teaching depends on the presence of both the professor, the student and a clinical case, it cannot be scheduled and thus asynchronous interaction could be an innovative way for students to make the most of their rotations.

In our study, we show that setting up accounts with limited privileges for students in the CPOE system enabled us to try a new method of teaching and to conduct this trial with no modification on the existing infrastructure. It is a proof of concept that this method can be implemented throughout the whole hospital without any real difficulty regarding the hospital management or IT implementation standpoints. Furthermore, it readily allows for a routine monitoring of the quality of prescriptions by the students, and shows that utilization of CPOE by the students can be used to refine teaching practices. This would make possible follow-up studies on order behavior and patient care and see how students (and their CRs) differ in their practice and how this impacts on the transmission of knowledge. These different prospects are examples of how IS/IT (here the CPOE system) can be leveraged to quickly and easily implement, test and roll out new policies hospital-wide with virtually no overhead.

The acceptance of CPOE and of its benefits seems to be unrelated to typing proficiency or computer literacy. The main concerns are about patient security and confidentiality, and time devoted to computer use. Ease of use, integration in the workflow and mostly training are seen as factors in their acceptance and approval.[5,20] As the opinion about CPOE/EHR follows a declining trend with the medical training[4–6], and with the push for the use of EHR in hospitals and for general practitioners, it is logical to train medical students as early as possible to use these tools. In this course of action, evaluating the (eventually positive) side effects in terms of medical learning had to be done, when concerns about losing relational skills and contact with the patient are starting to appear.[21]

## Conclusions

We conducted an educational randomized controlled trial with a cross-over design assessing the impact of the use of CPOE at the bedside by medical students on their training, satisfaction with patient care, and involvement in the medical team. In line with the handful of previous trials addressing the same issue, we found no statistically greater improvement in the students’ ability to place adequate monitoring and therapeutic orders in the group using CPOE. We tried to address as much caveats from the previous trials as possible, but our study has got its own shortcomings, statistical power being the main one. However, whereas we confirm that CPOE use does not allow a better acquisition of prescribing skills, it is notable that it improves students’ satisfaction. Not only does it so in regard to the tool used, but also in the larger scope of patient care and feeling like being part of the medical team. Students are also able to place admission and follow-up orders when using the CPOE, thus inherently being a training in the use and master of the computer tool.

As the future is shaped towards a universal use of CPOE tools in hospitals, and with the evidence that first familiarization with these tools gets harder with professional experience, we recommend the introduction of computerized order entry as soon as possible in medical students training during their clerkship rotations.

## Supporting Information

S1 Table Data for the Short open answers questionnaire. (CSV)

S2 Table Data for the Satisfaction questionnaire. (CSV)

S3 Table Prepared data for the short open answers questionnaire. (CSV)

S1 Text Short open answers questionnaire, translated from French. (DOCX)

S2 Text Satisfaction questionnaire, adapted from Knight et al. (DOCX)
