# Supplementary material for: Evaluating the Impact of Computerized Provider Order Entry on Medical Students Training at Bedside: A Randomized Controlled Trial
Source: PLoS One. 2015 Sep 14;10(9):e0138094. doi: 10.1371/journal.pone.0138094 (PMC4569055; doi:10.1371/journal.pone.0138094)
Supplement: S1 Text — (DOCX) [file pone.0138094.s004.docx]

Appendix 1: Short open answers questionnaire

Part I

1 The chemistry panel of a patient taken that morning reveals a 2.7 mmol/l hypokalemia. You decide to supplement intra venous; he is already infused with a venous line. What is you prescription? (2 points)

2 When do you check serum potassium again? (2 points)

3 In front of a 6.3 mmol/l acute hyperkalemia without electrical symptomatology, you decide to administer insulin and glucose. What is your prescription? (2 points)

4 A patient receiving 1 mg/kg/day of corticosteroids has a 1.5 g/l fasting glucose. You decide to prescribe a rapid insulin protocol (eg NOVORAPID). Provide details. (4 points)

5 A patient with a lung transplant takes immunosuppressants (CELLCEPT & PROGRAF) at 7am and 7pm. You are asked to check his residual Prograf the next day. At what time do you check it? (1 points)

6 You are on call for emergencies. At 11pm, a patient comes in with sepsis; the call point is urinary. Prescribe first line complementary examinations. (4 points)

7 The blood test that you get after 2 hours shows an inflammatory syndrome and marked leukocytosis without other abnormalities. The patient is hyperalgic; the pain is localized in the lumbar pit and radiates forward. You decide to perform a renal ultrasound. Write your request. (2 points)

8 The ultrasound is normal. Upon reflection, you decide to initiate antibiotic therapy with CLAFORAN. Write your prescription. (1 points)

9 In the absence of complications, how long will the antibiotics prescription last? (1 points)

10 A patient comes to the emergency room with respiratory distress. Thanks to your care, the situation stabilizes quickly. What monitoring do you prescribe? (3 points)

11 You diagnose pulmonary tuberculosis in a 60kg patient with no history. What molecules do you prescribe? (1 points)

12 Provide details for your prescription. (3 points)

13 What do you watch out for? (3 points)

14 What is your first line check-up in an HIV-infected patient with 90 /mm3 CD4 and a 39°C fever? (6 points)

15 With a strong suspicion of nosocomial pneumonia, you decide to prescribe vancomycin and tazocilline. Provide details. (2 points)

16 What prophylactic treatment do you prescribe to an HIV positive patient with less than 200 /mm3 CD4? (1 points)

17 In case of allergy what are the other options? Details? (2 points)

Part II

1 Which platelet aggregation inhibitor would you prescribe (with dosage, duration of treatment) for a 78 years old patient hospitalized for acute coronary syndrome (ACS)? (4 points)

2 What are the additional first-line tests to be prescribed for a patient presenting to the ER with chest pain? (5 points)

3 What are the precautions to take before installing a pacemaker? (5 points)

4 What are the two classes of medication to prescribe if the patient is allergic to iodine before coronary angiography? (2 points)

5 What are the two main indications of ivabradine? (2 points)

6 What are the three ischemia tests (with a locative value) available for a patient with stable angina? (3 points)

7 Ms. V is treated with vitamin K antagonists (Previscan 1 tablet per day) for deep vein thrombosis. She calls you a morning as she just completed her biological analyzes. Her INR is 3.8. She asks you what to do. What do you tell her? There is no concept of bleeding. (2 points)

8 What laboratory tests do you prescribe to monitor the safety and efficacy of statin therapy? (3 points)

9 Mr T. is hospitalized for a well tolerated 152 bpm supra ventricular tachycardia (atrial fibrillation). There is no major history whatsoever. What treatment(s) do you prescribe (with doses)? He weighs 82 kg. (3 points)

10 Mr X is effectively anti-coagulated with heparin in a continuous action syringe for pulmonary embolism. How and how often do you watch his treatment? (5 points)

11 What tests do you prescribe a 72 years old patient waiting for an aortic valve replacement as part of his preoperative evaluation? (5 points)

12 What Atorvastatin dose do you prescribe to a young patient with STEMI? (1 points)

13 What dose of rosuvastatin do you prescribe to a young patient with STEMI? (1 points)

14 What treatment do you recommend (with dose and duration) for the treatment of pericarditis in a 24 years old patient with no history? (3 points)

15 You see Mrs Y in consultation for essential hypertension. You decide to prescribe amlodipine. With what dose do you start? (1 points)

16 What platelet aggregation inhibitor do you prescribe the eve of a scheduled stable angina angioplasty? (2 points)

17 Ms Z. is hospitalized in intensive care for a complete A-V block with a poorly tolerated ventricular escape oscillating between 35 and 35 bpm. What do you prescribe as first-line drug therapy? What do you suggest in case of inefficiency? (2 points)

18 Mrs. B., 63, has a history of double valve replacement (aortic and mitral) with two mechanical valves. She is effectively anticoagulated with vitamin K antagonists (Previscan 1/2 tablet per day). Her INR today is 1.9. What drug therapy do you prescribe? (5 points)

19 What are the four imaging tests ordered before percutaneous aortic valve replacement? (4 points)

20 What drug do you prescribe in case of cardiogenic shock and with what dose (specify the therapeutic objective)? What precautions do you take? (4 points)

Part III

Observation I

Mr. X, 83, is hospitalized for delirium with 120 mml/l hyponatremia. On admission he is normotensive, and shows no signs of fluid overload. His medical history includes: hypertension for 20 years, depressive syndrome, ischemic stroke in 2010 and acute coronary syndrome in 2008. His treatment includes: COAPROVEL, PAXIL, ASPEGIC, and DETENSIEL.

1 What treatment do you prescribe for the first 24 hours? (2 points)

2 What control do you program and how often? (4 points)

3 Which first line exam do you ask to find the cause of hyponatremia and when? (2 points)

4 After 3 days of saline infusion, serum sodium is 123 mmol/L, serum potassium 4.6 mmol/l, glucose 7 mmol/l, urea 7 mmol/l, creatinine 90 mmol/l, urinary electrolytes shows Na = 90 mmol/l, K = 35 mmol/l, urea 125 mmol/l, creatinine 40 mmol/l. What are the three differential diagnoses and what examinations do you prescribe to find the right one? (5 points)

5 What treatment do you prescribe, while waiting for the results? (1 points)

Observation II

Mr. Z, 58, has been hospitalized for fever for 48 hours. His main medical history is follicular lymphoma for which he received six months of chemotherapy with R-CHOP on central catheter. His last chemotherapy was 10 days ago. There is no clinical sign of infection. His last blood test, performed two days earlier shows: Leukocyte 2500 / mm3, neutrophilic leukocyte 1050 / mm3, Hemoglobin = 8.1gd / L, MCV = 95 fl, platelets = 70 000 / mm3

6 What tests do you begin with? (4 points)

7 The blood cell counts found 900 leukocytes including 400 neutrophilic leukocytes / mm 3, anemia with 7.8 g / dL hemoglobin and 30 000 platelets / mm3. The blood ionogram shows: Na 130 mmol / l, K 5 mmol / l, creat 180 umol / l. What treatment do you start and within which delay? (4 points)

8-What additional tests do you urgently ask to explore kidney failure? (3 points)

9 The patient is no longer in aplasia, and without infection found the treatment is halted. Kidney failure persists, and it was actually already present six months ago at diagnosis of lymphoma (it is found in the hematology records). However, after 7 days the patient starts to be febrile again and exhibits a painful, big red hot right knee. What complementary examination do you prescribe? (4 points)

10- Findings deem gout: what treatment do you begin and for how long? (3 points)

Observation III

Miss K, 20, with sickle cell SS, is hospitalized for a painful crisis localized in the 2 legs for 48 hours, not giving in to level 2 painkillers. Her hemoglobin is usually at 8 g / dl. She endures 2-3 vasoocclusives serious crises per year and has already been hospitalized once in the ICU, she had no previous surgical history. She is afebrile.

11 What tests do you begin with? (5 points)

12 Leukocytes are at 10 500 / mm3, hemoglobin is 7.7 g / dl, platelets are at 450 000 / mm3. The results of other tests ordered are normal or expected. What treatment do you prescribe? (5 points)

13-These tests are normal, and the next day the pain is localized at the right base of the thorax and the patient becomes dyspneic (RR 25 / min) with a 92% oxygen saturation on 2 liters of O2 / min. Chest auscultation is normal. What emergency tests do you prescribe? (3 points)

14 The results make you fear a pulmonary embolism. How you confirm the diagnosis? Do you modify her treatment before obtaining the results? (3 points)

15 Pulmonary embolism is confirmed, what treatment do you start on and how do you watch? (5 points)

Observation IV

16 Mr A, 78, presents a macrocytic anemia (Hb 9 g / dl and MCV 110 fl). He claims drinking alcohol only occasionally. What first line noninvasive tests do you prescribe? What other examination do you prescribe if they are not contributing? (4 points)

17 In fact, his wife tells you that he drinks two glasses of wine per meal ("it's not alcohol") plus two or three beers and liquor in the evening... After 24 hours hospitalization he becomes tremulous, with a 37.9°C low-grade fever, 120 / min tachycardia, 16/9 hypertension and is agitated: you diagnose a withdrawal syndrome. What treatment do you prescribe? (3 points)

18 The patient is hospitalized again a year later for the onset of edema of the lower limbs and ascites for a week. Blood cells count again finds a 9 g / dl macrocytic anemia, serum sodium is 128 mmol / L, serum potassium and creatinine are normal. Transaminases are at 3 times the normal values, gamma GT 10 times and Alkaline phosphatases 2 times. What complementary examination do you prescribe on arrival? (5 points)

19 The results confirm the diagnosis of cirrhosis but do not find the trigger to the ascites decompensation. What treatment do you start and how do you modify it if it is not effective? (4 points)

20 How do you watch? (5 points)
