# Supplementary material for: Evaluating the Impact of Computerized Provider Order Entry on Medical Students Training at Bedside: A Randomized Controlled Trial
Source: PLoS One. 2015 Sep 14;10(9):e0138094. doi: 10.1371/journal.pone.0138094 (PMC4569055; doi:10.1371/journal.pone.0138094)
Supplement: S2 Text — (DOCX) [file pone.0138094.s005.docx]

Appendix 2: Satisfaction questionnaire adapted from Knight et al (2005)

Part I: Attitude toward placing orders

On the following scale: "Strongly Disagree", "Disagree", "Neutral", "Agree", "Strongly Agree"

1) Placing orders is an important way to increase my sense that I am a caregiver for my patients

2) Placing orders is an important way to learn what tests and treatments are needed by patients with certain problems

3) Entering orders by computer promotes ‘‘cookbook medicine’’ and discourages thinking

4) Writing orders by hand is cumbersome

5) Writing orders by hand encourages medical errors

6) Entering computerized orders is cumbersome

7) Entering computerized orders encourages medical errors

8) It makes no difference in my learning whether I enter orders by computer or write them on paper

9) Medical students should be given as many opportunities as possible to place orders for their patients

10) The ordering method used will have an impact on my selection of the location of my future rotations

11) The ordering method used will have an impact on my selection of the location of my residency

Part II: Patient care ability

On the following scale: "Bad", "Fair", "Good", "Very good", "Excellent"

1) During this rotation, what was, in your opinion, your contribution to the medical care of patients?

2) During this rotation, what was, in your opinion, your contribution to the prescription for a patient (paper or computerized prescription)

Part III: Satisfaction

Likert scale: "Strongly Disagree", "Disagree", "Neutral", "Agree", "Strongly Agree"

1) I felt like part of the medical team in the care of my patients

2) I was included in discussions about the management of my patients

3) My intern and resident thought it was important for me to have opportunities to place orders on my patients

4) My chief resident thought it was important for me to have opportunities to place orders on my patients

5) My other seniors (other than chief resident or resident) thought it was important for me to have opportunities to place orders on my patients

6) I am receiving adequate training in how to write/enter orders

7) I am receiving adequate preparation for being an intern

Part IV: Barriers to placing orders

Scale: "No", "A little", "Average amount", "A lot"

1) Resident or intern did not want me to write or enter orders

2) Chief resident did not want me to write or enter orders

3) Other seniors (other than chief resident or resident) did not want me to write or enter orders

4) It took too long for the resident or intern to review the orders I wrote

5) It took too long for the chief resident to review the orders I wrote

6) It took too long for other seniors (other than chief resident or resident) to review the orders I wrote

7) Difficulty in finding a free computer terminal

8) Resident, intern, chief resident or other seniors did not know how to electronically cosign my orders

9) Inadequate training on the computer ordering system

10) Computer ordering system difficult to use

Part V: Preferences about order placement and review

1) For what percent of newly admitted patients that you picked up have you entered the complete set of admission orders?

2) What percentage of your patients’ total number of follow-up orders would you like to write or enter?

3) What percentage of your patients’ total number of admission and follow-up orders would you like to review with:

a. your resident?

b. your chief resident?

c. another senior?
